# *Vibrio metschnikovii*: Current state of knowledge and discussion of recently identified clinical case

**DOI:** 10.1002/ccr3.3999

**Published:** 2021-03-04

**Authors:** Yulian Konechnyi, Yurii Khorkavyi, Kateryna Ivanchuk, Ihor Kobza, Alicja Sękowska, Olena Korniychuk

**Affiliations:** ^1^ Department of Microbiology Danylo Halytsky Lviv National Medical University Lviv Ukraine; ^2^ Department of Surgery #2 Danylo Halytsky Lviv National Medical University Lviv Ukraine; ^3^ Ludwik Rydygier Collegium Medicum Nicolaus Copernicus University Bydgoszcz Poland

**Keywords:** case report, graft infection, rare pathogen, vascular surgery, *Vibrio metschnikovii*

## Abstract

*Vibrio metschnikovii* is a widespread opportunistic pathogen that rarely causes disease in human. It caused graft infection in our case. It is important to differentiate it from another water‐transmitted pathogens.

## Graphical abstract

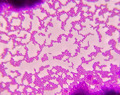

## 1 RELEVANCE

The literature review was done about *Vibrio metschnikovii*—a rare pathogen usually with water route of transmission. Thirteen clinical cases were described of *Vibrio metschnikovii* infection from literary sources and one clinical case, registered by us in patient after surgery due to aorto‐bifemoral alloprosthesis infection.

Due to extended globalization processes and climate changes, the humanity faces new and more alarming threats. These include new potential risk factors from opportunistic microorganisms, as well as epidemiological changes, especially in the healthcare environment. Nowadays, healthcare providers face unique and unusual pathogens that were not commonly causing diseases.

## 2 CASE REPORT

The 70‐year‐old male patient was hospitalized urgently in October 2019 into the intensive care unit of the hospital, situated in Lviv, Ukraine, suspected with upper gastrointestinal bleeding caused by the ulcer. Intestinal bleeding was managed conservatively. On the 3‐rd day after hospitalization, patient with posthemorrhagic anemia was transferred to the surgical department.

According to patient's medical history: 1991—bifurcated aorto‐bifemoral alloprosthesis placing due to Leriche syndrome; 1999—replacement of bifurcated aorto‐bifemoral prosthesis due to thrombosis; 2001—thrombosis of the right branch of bifurcated aorto‐bifemoral prosthesis, extra‐anatomical axillofemoral bypass performed with early thrombosis of reconstruction which led to limb amputation. After few years, femoropopliteal autogenous venous bypass was made due to chronic limb ischemia on the other side. At the time of hospital admission, the femoropopliteal graft was occluded without chronic limb‐threatening ischemia.

Clinical symptoms of gastrointestinal bleeding relapse were observed on the fifth day of treatment in the hospital. Though no source of bleeding was found during upper gastrointestinal tract endoscopy, multislice spiral computed tomography (MSCT) was performed. MSCT angiography has shown pseudoaneurysm formation of central anastomosis of aorto‐bifemoral graft and aorto‐small intestine fistula formation (reason of gastrointestinal bleeding). Ultrasonography has shown an exudate around the alloprosthesis. Surgery is considered to be the best option. The extra‐anatomical axilofemoral bypass, exclusion of the central anastomosis aneurysm, removal of infected graft, and aorto‐digestive fistula repair have been performed under general anesthesia. The blood loss was about 2250 mL, and autologous blood reinfusion was used. The hemostasis system control was made using TEG and aggregometry.

Postoperative period was complicated with: renal dysfunction; hospital‐acquired bilateral pneumonia; partial, closed eventration without need of surgery; postoperative wound healing by secondary intention (treated with NPWT (negative pressure wound therapy)); and antibiotic‐associated diarrhea.

After the surgical removal, Vibrio metschnikovii was found in large quantities (about 108 colony‐forming units/mL) on the surface of the removed Dacron prosthesis. The antibiotic sensitivity test is displayed in Table 1. From the same fragment, Trichosporum spp was isolated in insignificant quantities (resistant to clotrimazole and susceptible to fluconazole).

**TABLE 1:** Antibiotic sensitivity test of *Vibrio metschnikovii* (this study), R—resistance, iS—intermediate sensitive, S—sensitive

N	Antimicrobial drug	
1.	Amikacin	S
2.	Amoxicillin	S
3.	Amoxiclav	S
4.	Ampicillin	S
5.	Ampisulbin	S
6.	Azithromycin	S
7.	Aztreonam	R
8.	Bacitracin S	R
9.	Benzylpenicillin	R
10.	Cefazolin	S
11.	Cefazolin	R
12.	Cefepime	R
13.	Cefotaxime	S
14.	Ceftazidime	R
15.	Ceftriaxone	R
16.	Cefuroxime	S
17.	Ciprofloxacin	S
18.	Clindamycin	S
19.	Colistin	R
20.	Co‐trimoxazole	R
21.	Doxycycline	S
22.	Erythromycin	S
23.	Fosfomycin	R
24.	Furazidine	S
25.	Furazolidone	S
26.	Gatifloxacin	S
27.	Gentamicin	S
28.	Imipenem	S
29.	Intestiphage (bacteriophage polyvalent)	R
30.	Levofloxacin	S
31.	Linezolid	S
32.	Meropenem	S
33.	Metronidazole	R
34.	Moxifloxacin	S
35.	Netilmicin	S
36.	Novobiocin	S
37.	Ofloxacin	R
38.	Oxacillin	R
39.	Piperacillin	S
40.	Pyophage (bacteriophage polyvalent)	R
41.	Rifampin	S
42.	Sisomycin	S
43.	Sulbactomax (ceftriaxone / sulbactam)	R
44.	Sulperazone	S
45.	Teicoplanin	R
46.	Tetracycline	S
47.	Tigecycline	S
48.	Tobramycin	S
49.	Vancomycin	S

Note

Blue color—metabolizes the substrate; Orange color—does not metabolize the substrate.

The patient's condition was significantly improved after treatment with meropenem (10 days), metronidazole (3 days), and fluconazole (7 days). After that, he was transferred to a simple ward. After 10 days of treatment, the blood culture examination has shown *S. piscifermentans* in small quantities (about 106 colony‐forming units/mL). According to the literature review, *S. piscifermentans* is not pathogenic. Also, the patient was stable (no signs of infection). In that case, we suppose that the mistake was made during a blood sample collection.

The patient was discharged from the hospital in good condition after 67 days of hospital treatment. In the next 6 months, patient underwent ventral hernia repair surgery and reported significant improvement in his condition.

## 3 METHODS

### 3.1 Bacterial isolation and growth conditions

The isolation and identification of pathogens were carried out in a certified microbiological laboratory of the Department of Microbiology at the Danylo Halytsky Lviv National Medical University, Ukraine. A bacteriological method was used to isolate a pure pathogen culture with blood agar, meat peptone agar, and Sabouraud agar.

### 3.2 Biochemical identification

The pathogen identification was performed using the MIKRO‐LA‐TEST NEFERM24 kit (Erba Lachema, Czech Republic) in five technical repetitions and using the corresponding codebook. CANDIDATEST 21 kit was used for *Trichosporon* spp identification.

### 3.3 Antibiotic sensitivity test

Antibiotic sensitivity test was measured using disk diffusion method with interpretation according to genus *Vibrio* ^1^, ^2^ (Table 1).

## 4 DISCUSSION

*Vibrio metschnikovii* is a rare human pathogen, which could be classified as a partially zoonotic microorganism with the fecal‐oral route of transmission. Bacteria is ubiquitous, does not have an exact geographical location. From 1981 to 2014, there were 13 clinical cases of *Vibrio metschnikovii* infection reported and none from 2014 to 2020 (according to PubMed database). *Vibrio metschnikovii* infection could be fatal, but there was only one death out of 14 cases. The majority of patients were elderly people with long‐term concomitant diseases, which lead to secondary immunodeficiency, for example, diabetes, chronic heart failure, and chronic kidney failure.

Patients denied having contact with the sea or consuming marine products, so it was impossible to set the infection source in all of the cases.

The vascular prosthesis infection may be a result of the not perfect aseptic regime and bacterial contamination during surgery. Also, bacterial translocation to the vascular graft may occur from wound infection or intracavity abscess or due to the formation of erosion or fistula between the digestive tract and the graft. The less common cause is transient or chronic bacteremia, which can bring about the transmission of infection to synthetic materials in the body, especially in the early postoperative period when the endothelialization of the graft is not complete. Other surgical procedures near the vascular graft or paravasal thrombus inflammation with bacterial translocation also may be the reason for vascular graft infection.

We believe that vascular alloprosthesis, in our case, was infected through the aorta‐digestive fistula. But, it is important to mention a couple of genus *Aeromonas* pathogens selected from the intensive care unit of the same hospital within a month. The *Aeromonas* spp. were extracted from blood and urine and had infectious characteristics connected with providing medical care (the beginning of infection after hospitalization, multidrug resistance to antibiotics, others). It is known that *Aeromonas* spp. generally have water routes of transmission and are similar to genus *Vibrio,* according to some abilities. That is why these two types of bacteria are sometimes differentiated wrongly.^3^ In 3 months, there was selected one more species from another patient of this department. It was similar to *Vibrio* spp. conforming to its colony growth and microscopic figure. The surgery was done due to Takayasu disease, and the biopsy from the artery was taken, which revealed the pathogen in a low amount. The antibiotic therapy was not prescribed because the amount of the pathogen was not high enough, and there were no signs of infectious process.

*Trichosporon* spp. might be the reason for arterial vascular prosthesis infection,^4^ but in over case, *Trichosporon* spp. was selected in a low amount. So, it cannot be the leading cause of infection.

**FIGURE 1:**
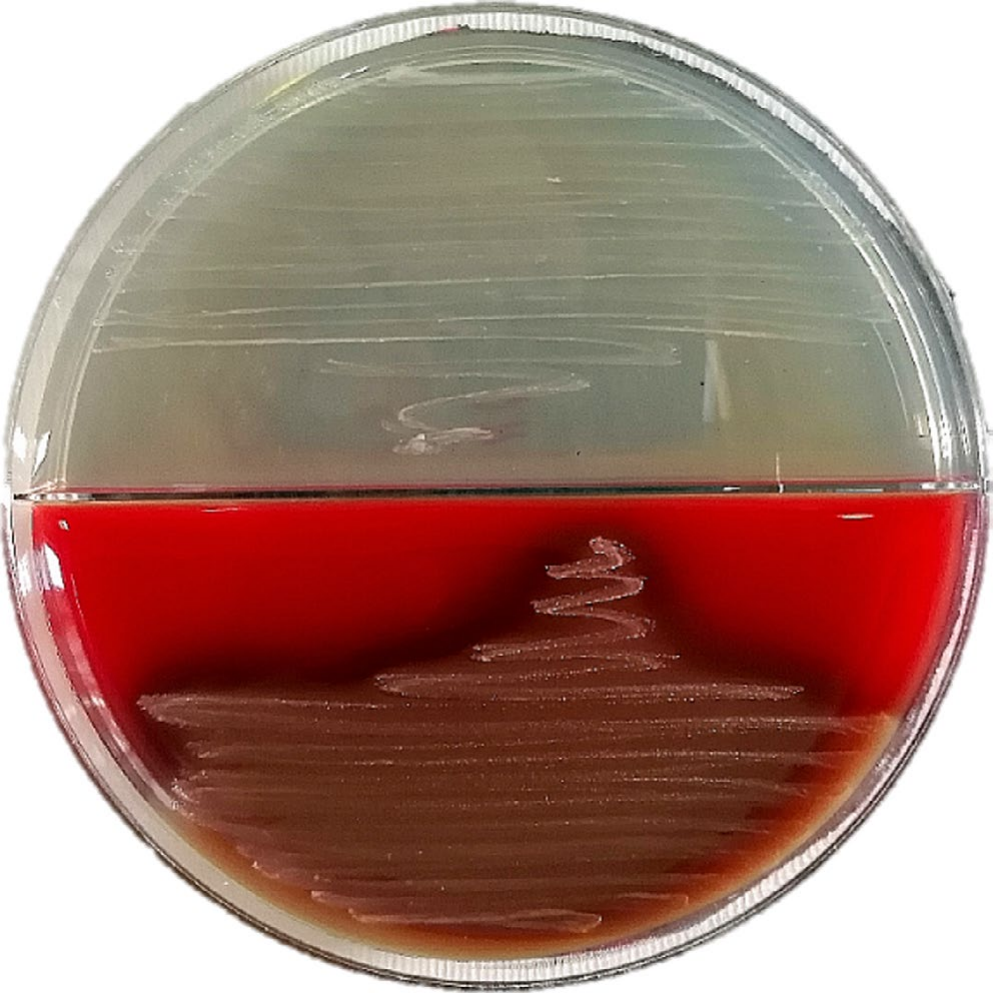
*Vibrio metschnikovii* stock culture on blood agar (below) and simple meat peptone agar (above), using streak plate technique for isolate a single colony

**FIGURE 2:**
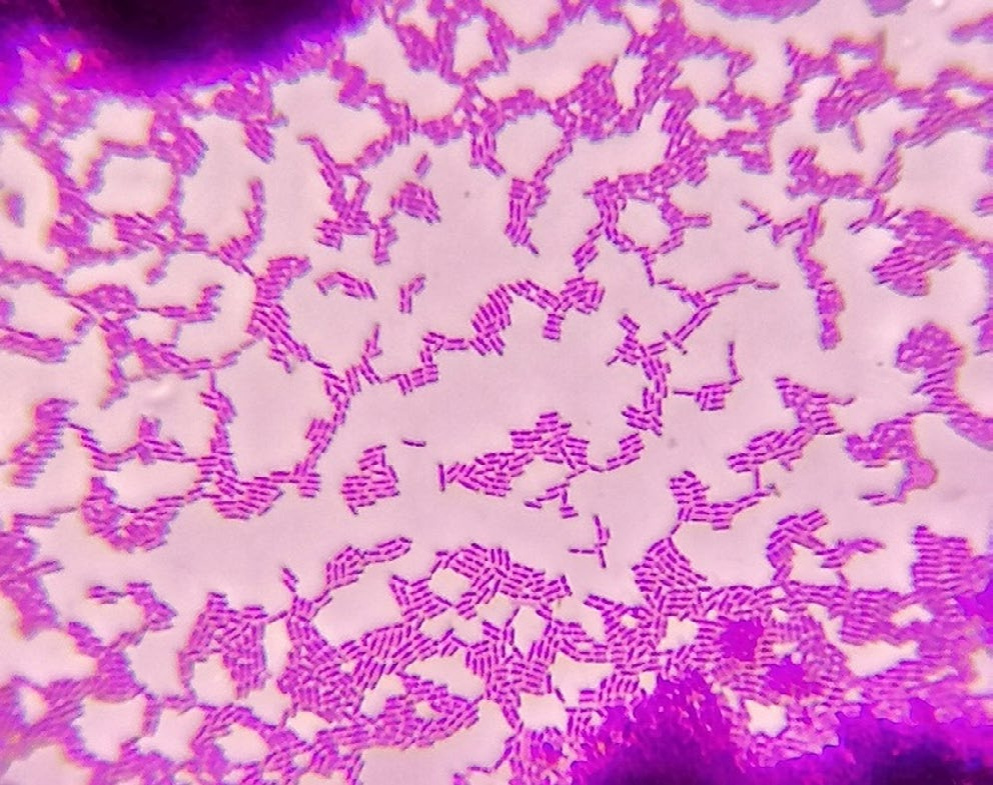
Light microscopy of *Vibrio metschnikovii* (Gram staining, 10 × 100 magnification). Gram‐negative rod‐shaped motile bacteria, 1‐2 μm, typically for *Vibrio* genus

In our case, *Vibrio metschnikovii's* cultivation is simple: on blood agar, with the temperature up to 37°C, causes beta‐hemolysis and does not have a specific smell or pigment. Colonies are nonspecific, and size is approximately 0.3 mm. (Figure 1). Initially, the pathogen looked like micrococci and only after passaging changed to rod‐shaped bacteria, with slightly bent ends (Figure 2). Biochemical characteristics were the same as described in literature.^5^ The result of biochemical identification is shown in Table 2.

**TABLE 2:** Optimal growth conditions and biochemical properties of *Vibrio metschnikovii* (this study)

Biochemical properties profile																		
OXI	URE	LAC	GAL	ARG	MAN	MLT	ORN	TRE	CEL	LYS	XYL	SUC	AAM	ARA	INO	BGL	AGA	GGT
																		
NAG	BGA	PHS	SCI	MAL	ESL	IND	PHE	VPT	H2S	ONP	SOR	ADO	RHA	MLB	RAF	DUL	MAL	GLU
																		

Note

Blue color—metabolizes the substrate; Orange color—does not metabolize the substrate.

Abbreviations: AAM, acetamide utilization; ADO, acid from adonitol; AGA, α‐glucosidase; ARA, acid from L‐arabinose; ARG, arginindihydrolase; BGA, β‐galactosidase; BGL, β‐glukosidase; CEL, acid from cellobiose; DUL, acid from dulcitol; ESL, aesculin hydrolysis; GAL, acid from galactose; GGT, γ‐glutamyle‐transferase; GLU, acid from glucose; H2S, hydrogen sulfide production; IND, indole production; INO, acid from myo‐inositol; LAC, acid from lactose; LYS, lysindecarboxylase; MAL, malonate utilization; MAL, malonate utilization; MAN, acid from mannitol; MLB, acid from melibiose; MLT, acid from maltose; NAG, N‐acetyl‐glucosaminidase; ONP, β‐galactosidase (ONPG); ORN, ornithindecarboxylase; OXI, oxidase; PHE, phenylalanindeaminase; PHS, phosphatase production; RAF, acid from raffinose; RHA, acid from rhamnose; SCI, citrate utilization; SOR, acid from sorbitol; SUC, acid from saccharose; TRE, acid from trehalose; URE, urea hydrolysis; VPT, acetonin production; XYL, acid from xylose.

In all described cases were no multidrug‐resistant strains. Moreover, there were no similar cases with similar antibiotic resistance. For example, some of them were resistant to ampicillin; otherwise, some were susceptible to it. It could be classified as an exogenous (more iatrogenic) opportunistic infectious factor.

### 4.1 Literature review

Genus *Vibrio* belongs to the family *Vibrionaceae*, class Gammaproteobacteria and includes 140 ubiquitous species. These bacteria are more common in the salty and freshwaters all over the planet.^6^ It lives as plankton, associated with free chitinous material, algae. 13 species of genus *Vibrio* are associated with human diseases, it causes diarrhea or extraintestinal symptoms (wound and skin infections, bacteremia), particularly *V. cholerae*, *V. parahaemolyticus*, and *V. vulnificus* consider to be the most significant. Water routes and uncooked marine products are the main ways of transmission. It is common for genus *Vibrio* to cause illnesses of fish (especially trouts), shellfish, eels, and oysters. For example, eels and fish (rainbow trouts and salmonidae) could have terminal hemorrhagic septicemia caused by *V. anguillarum*; sea perches—gastroenteritis caused by *V. harveyi*; larvae of flounders—intestinal necrosis caused by *V. ichthyoenteri* and Japanese horse mackerel—intestinal hemorrhage caused by *V. trachuri*.^5^

*Vibrio metschnikovii* is ubiquitous in water (both salty and fresh), particularly in 2020, 38 species of *Vibrio metschnikovii* were selected from the Norwegian Sea nearby the Norwegian coast and from the herring fish of the same Sea.^7^ *Vibrio metschnikovii* is associated with the ability to cause human diseases, mostly diarrhea, skin infections, and bacteremia; otherwise, some authors say that *Vibrio metschnikovii* induce only extraintestinal symptoms. Mostly, the pathogen can be isolated from blood, urine, wound surfaces, bile, and feces. In general, it infects fish, particularly sardines that have their gills, digestive system, and ovaries in females affected (despite other *Vibrio* spp), with testicles in males unaffected.^8^

*Vibrio metschnikovii*, the same as other species of genus *Vibrio,* belong to halophiles (organisms that thrive in high salt concentrations). It is common for vibrio to decrease in size and lose its volume (15%‐67%), changing from rod‐shaped to cocci (spherical ultramicrococci) when they are in a lack of nutrients. They are mobile, mostly oxidase‐negative (due to cytochrome C insufficiency, but some are oxidase‐positive), Gram‐negative, facultative anaerobic rods.^9^ The main pathogenic factors are hemolysin (*tlh* gene), some genes of toxic formation are present (*rtx*A gene, responsible for producing holotoxins). The ability to secrete cytotoxin was confirmed when *Vibrio metschnikovii* was indicated in aborted fetuses of pigs, cattle, and horses, moreover in different parts of the duck's and geese's brain in Germany. Also, this pathogen was found in the chicken's intestines and together with *E. coli* caused hepatitis.^10^ 38 strains indicated nearby the Norwegian seashore had the genes of resistance adeF (responsible for polypharmacological efflux mechanism), blaCARB (responsible for carbenicillin resistance), catB‐related (responsible for chloramphenicol resistance), and genes that are responsible for pathogenicity factors Hem *III* (hemolysin type III), *hylA, tlh*.^7^ Usually, the pathogen has huge plasmids (190‐340 кB), but their function is not exactly known.

Ten strains were isolated from the lakes, specifically those situated on the graphite quarries, in Slovakia had 100% resistance to penicillin and 90% streptomycin resistance.^6^ 13% pathogens found in the Norwegian Sea turned out to be tobramycin‐resistant and 21%—ampicillin‐resistant.^7^ Some authors^5^ report about 91% resistance to penicillin G, 95% sulfadiazine, 86% kanamycin, and 69% ampicillin resistance.

Due to the ecological plasticity of the *Vibrio metschnikovii*, the cultivation is relatively simple. The optimal conditions are as follows: pH—6.0 (but can vary up to 10.0); temperature—30‐40°C; NaCl concentration—1.0%‐8.0%. The best culture media are as follows: Sheep blood agar, Alkaline peptone water, Marine broth, Marine agar, Photobacterium agar, Photobacterium broth, Selective media for Vibrio such as thiosulfate‐citrate‐bile salts‐sucrose agar (TCBS) and Vibrio agar; MacConkey agar not a priority for *Vibrio* cultivation.^5^ *Vibrio metschnikovii* causes RBC's hemolysis in vivo and in vitro (alpha‐ and beta‐hemolysis on blood agar). Isolation of the pathogen is more frequent in warm seasons of the year (typically May‐August). *Vibrio metschnikovii* is less susceptible to the cold environment in comparison to other *Vibrio* spp.

*Vibrio metschnikovii*, together with *Vibrio gazogenes,* are unique species of genus *Vibrio*. They are nitrate negative (unable to convert nitrates to nitrites) and oxidase‐positive (some strains). Moreover, these two species differ from other *Vibrio* spp. by their immunological relation to superoxide dismutase. Finally, it is essential to mention that results of DNA‐DNA hybridization may be the reason for further reclassification into separate genus.^5^

### 4.2 Review of case reports

There are 13 clinical cases of *Vibrio metschnikovii* infections, according to the PubMed database.

The first clinical case was described in 1981.^11^ 82‐year‐old woman was diagnosed with acute cholecystitis that led to peritonitis. After this case, river and wastewaters, mussels, shrimps, lobsters, crabs, and poultry were considered to be infectious factors. Besides, 5 clinical cases of acute watery or hemorrhagic diarrhea in infants were recorded in Peru.^12^ The first description of postoperative wound infection with *Vibrio metschnikovii* was made in 2004. It was a 64‐year‐old male patient who underwent bilateral saphenectomy combined with cardiac surgery.^10^ All these cases did not include multidrug‐resistant strains of *Vibrio metschnikovii*. Another interesting case was septic shock and cardiac arrest in a 78‐year‐old Dutch male caused by *Vibrio metschnikovii* in 2014.^13^ A short review of all clinical cases is shown in Table 3.

**TABLE 3:** Review of all reported cases of Vibrio metschnikovii infection (published until 2020)

Author (ref)	Year	Sex/age	Country	source of infection	Co‐morbidity	Type of infection	Co‐infections	Maine clinical symptoms	Susceptible to	Resistance to	Antibiotic treatment	Outcome
11	1978	F/82	USA	N/A	Acute cholecystitis and ascending cholangitis) б diabetes mellitus controlled with diet and hypertension treated with diuretics.	Bacteremia	N/A	Vomiting, weakness, chills, diarrhea, abdominal pain	N/A	N/A	Tobramycin and clindamycin	Treated
14	1992	F/83	Norway	N/A	Myocardial infarction	Bacteremia	*Staphylococcus hominis*, *E. coli*	Chest pain, remained unconscious, high fever, chills, and malaise	Ampicillin, cephalosporins, fluoroquinolones, and netilmicin, *Staphylococcus hominis* isolate did not produce beta‐lactamase and was susceptible to cephalosporins, clindamycin, vancomycin, and netilmicin	N/A	Ampicillin	Treated
15	1993	M/70	Belgium	N/A	Tabagism (smoking for more than 50 y), alcohol abuse, insulin‐dependent diabetes, renal insufficiency, alcoholic cirrhosis, blood‐clotting abnormalities, and a duodenal ulcer.	Bacteremia	N/A	Type III dyspneic symptoms, weakness, abdominal pains, diarrhea, vomiting, nausea, vertigo, and headache	N/A	N/A	N/A	Died of myocardial infarction
15	1993	F/82	France	N/A	Emphysema, asthma, cutaneous leg ulcers, cardiac insufficiency for the past 2 y, and hypertension	Bacteremia, swab samples of the patient's leg lesions	*Vibrio metschnikovii* in blood and *Vibrio metschnikovii* + mix (Morganella morganii, Serratia marcescens, Enterococcus faecalis, Klebsiella pneumoniae) in swabs from her leg ulcerations and pleural effusion	Severe dyspneic problems, significant weakness, and serious cutaneous lesions of the lower limbs.	N/A	N/A	Amoxicillin‐clavulanate, tobramycin	Treated
12	1994‐1995	Five infants 4 males/1 female/11‐20 mo old	Peru	N/A	N/A	Stool specimen or rectal swab	N/A	Acute diarrheal disease, including frequent passage of liquid or semi‐liquid stools, two patients had diarrhea with traces of blood.	N/A	Ampicillin, erythromycin, and streptomycin	N/A	N/A
10	2004	M/64	Germany	N/A	Cardiac surgery	Postoperative wound infection	N/A	Signs of local inflammation included erythema and discharge of exudate after pressure but no pain	Mezlocillin, piperacillin, piperacillin‐sulbactam, carbapenems, expanded‐ and broad spectrum cephalosporins, aztreonam, fluoroquinolones, trimethoprim, fosfomycin, tetracycline, and aminoglycosides	Ampicillin and sulfamethoxazole	N/A	Treated
16	2005	M/63	France	N/A	Chronic obstructive pulmonary disease	Pneumonia	Nonhemolytic streptococci	Acute respiratory failure related	N/A	Ampicillin, ticarcillin, piperacillin, and aminoglycosides.	Amoxicillin/clavulanic acid, ciprofloxacin	Treated
17	2008	F/49	Spain	N/A	Fibromyalgia and frequently infected leg ulcers	Infected leg ulcers	N/A	Painful ulcers in both legs	Cefoxitin (MIC ≤ 8), amikacin (MIC = 16), amoxicillin/clavulanic acid (MIC ≤ 4/2), imipenem (MIC ≤ 1), ciprofloxacin (MIC ≤ 0, 12), and trimethoprim/sulfamethoxazole (MIC ≤ 2/38)	Amoxicillin (MIC > 16 µg/mL), cefalotine (MIC > 8), cefotaxime (MIC = 8), gentamycin (MIC > 8), tobramycin (MIC > 8	Imipenem and amikacina	Treated
13	2014	M/78	Danmark	N/A	Bipolar disorder, multiple myeloma, osteoporosis, earlier pituitary tumor, and development of frontotemporal dementia.	Bacteremia	N/A	Pain, swelling, and redness of his left dorsalis pedis, fever	Meropenem, piperacillin/tazobactam	Ampicillin	Meropenem, piperacillin/tazobactam	Treated
This study	2019	M/70	Ukraine	N/A	Heart failure, multifocal atherosclerosis, vascular prosthesis, chronic gastric ulcer, gastrointestinal bleeding, chronic kidney disease	Vascular prosthesis, bacteremia	*Trichosporon* spp from vascular prothesis	Recurrence gastrointestinal bleeding, abdominal pain, black stools, cough, shortness of breath	Tab.3	Tab.3	Meropenem, metronidazole, fluconazole	Treated

## 5 CONCLUSION

Nowadays, it is important to be aware of new pathogens, which commonly were not considered to cause an infection in humans. As new technologies of endoprosthesis develop, new measures to prevent the infection should be taken.

The use of antiseptics and hypertonic solutions during wound care is effective mostly for common pathogens that are in priority. On the other hand, they could be useless and purposeless for some species that were not tested and turned out to be resistant. This might happen due to the lowering of the human's body immune system resistibility and deterioration of the protective barriers, making it effortless for the pathogen to infect the body.

## CONFLICT OF INTEREST

All authors have no conflict of interests.

## AUTHOR CONTRIBUTIONS

YK: involved in writing and original draft preparation. YK: involved in main clinical part executant. KI: involved in visualization and translation. IK: involved in supervision. AS: involved in review and editing. OK: involved in project administration.

## ETHICAL APPROVAL

Protocol number 6 of Ethics Committee of research, experimental development and scientific works of Danylo Halytsky Lviv National Medical University (Ukraine) behalf of 25 June 2018.

## Data Availability

The information used and/or analyzed during this case report is available from the corresponding author on reasonable request. If requested (please contact yulian.konechnyi@gmail.com ).
